# “I was bullied for being fat in every situation, in every outfit, at every celebration”: A qualitative exploratory study on experiences of weight-based oppression in Qatar

**DOI:** 10.3389/fpubh.2023.1015181

**Published:** 2023-02-27

**Authors:** Lily O'Hara, Bayan Alajaimi, Bayan Alshowaikh

**Affiliations:** Department of Public Health, QU Health, Qatar University, Doha, Qatar

**Keywords:** weight stigma, weight-based oppression, weight bias, Arab region, Qatar

## Abstract

**Introduction:**

Weight-based oppression (WBO) has been documented as a widespread phenomenon in Western countries and is associated with a range of psychological, physiological, and behavioral harms. Research on weight-based oppression is largely absent from the Arab region.

**Methods:**

We conducted a qualitative exploratory study using semi-structured in-depth interviews to examine the internalized attitudes, values, and beliefs related to body weight, and experiences of external weight-based oppression of 29 staff, faculty, and students at Qatar University.

**Results:**

Thematic analysis revealed six major themes on the characteristics of internalized WBO, and the nature, timing, source, extent, and impact of external WBO. WBO was regarded as so common in the Arab culture as to be normative, with damaging exposure to WBO beginning in early childhood.

**Conclusion:**

WBO in the Arab region is an important and unrecognized public health issue. Programs to reduce WBO should be developed in all sectors.

## 1. Introduction

Weight-based oppression (WBO), including negative beliefs, teasing, harassment, stigma, prejudice, and discrimination based on body weight, is a widespread phenomenon that leads to considerable distress, health harming behaviors and poor health outcomes (1). WBO arises from both external and internal sources. External sources of WBO include exposure to stigmatizing or exclusionary social, cultural, economic, political, and built environments, weight bias and discrimination, and weight-based bullying and violence. In Western countries, the prevalence of weight stigma is high, and appears to be increasing (2). Up to a half of young people in the USA have been subjected to weight-based harassment, the highest rate of any type of harassment, and similar to or greater than rates of ethnicity-based harassment (3, 4). Up to one third of young people have been subjected to weight based discrimination (4). Weight-based harassment is prevalent across genders, with up to 65% of non-binary and transgender youth experiencing weight-based victimization (4). Those with higher weights, women, transgender, non-binary, queer, and younger people are subjected to the highest levels of weight-based discrimination (4–6). Weight bias has been demonstrated to exist across all aspects of society including in health care practitioners, educators, employers, landlords, and the general public (7).

WBO from external sources is linked with decreased body satisfaction, lower self-esteem, greater weight concerns, more loneliness, higher depressive symptoms, suicidal thoughts and attempts, higher preference for sedentary activities or activities performed alone, and bulimic behavior, regardless of actual body weight (8). Mental health issues such as depression are associated with being exposed to weight-based teasing, bullying and stigmatization. These associations are apparent across gender, ethnic, racial, and weight groups (9). The more sources of teasing that people are exposed to, the greater the prevalence of emotional health issues. In one study, participants that were exposed to teasing from their peers had considerably higher levels of depression and were five times more likely to adopt health harming weight manipulation behaviors compared to those who were not exposed to teasing (10). The frequency of teasing and the number of teasing sources significantly increased the risk of depression. Weight-based teasing strongly predicts binge eating and intensive weight manipulation over 5 years (11). Negative comments from parents about weight or shape and eating are associated with psychological distress and eating disorder cognitions in adolescents (12). A systematic review found an association between weight teasing by parents and problematic eating behaviors in adolescents (13).

Internalized WBO is the negative attitudes, values, and beliefs people hold about one's own weight (14), which have negative consequences for health and wellbeing (15). Internalized negative attitudes about body weight are so strong that being fat is considered worse than having breast cancer (16), and a proportion of people would rather lose a limb, be blind, alcoholic, severely depressed, unable to have children, or lose 10 years of life or more than be fat (17). Internalized WBO is linked to low self-esteem, anxiety, depression, avoidance of physical activity, body image disturbance, decreased use of preventive health services, increased calorie consumption, disordered eating, and weight gain (18–21). Evidence is mounting of the psychological (22–27), behavioral (21, 25, 28–32) and physiological effects of WBO. Physiological effects include higher blood pressure (33, 34), type 2 diabetes mellitus (35), metabolic syndrome (36, 37), allostatic load (lipid/metabolic dysregulation, glucose metabolism and inflammation) (38), cortisol reactivity (39), and oxidative stress (40).

Research on external WBO is largely absent from the Arab world, including Qatar. The only study to address any aspect of external WBO found that 44% of female Emirati students reported being frequently teased about their weight, and that eating disorder symptomatology was positively correlated with being bothered by weight-based teasing and internalized weight stigma (14). Although there have been numerous studies in the Arab region on internalized WBO, they have tended to focus exclusively on body dissatisfaction and disordered eating attitudes [for recent examples see (41–45)]. A recent review of 22 studies involving over 10,000 adolescents from nine Arab countries found that the overall prevalence of disordered eating attitudes was 26.94%, a higher prevalence than in the USA or sub-Saharan Africa (46).

The “global culture of modernity” (47) has come to characterize many rapidly urbanizing parts of the world, including the Arab region, and is viewed as eliciting a rise in average body weight, weight consciousness, and disordered eating. Gordon (48) reviewed epidemiological data for nations where eating disorders first began being reported in the 1990s. He identified four pivotal characteristics that these nations had in common: (1) rising average body weights, (2) highly developed economies or rapid economic change, (3) changing and conflicting gender roles for women, and (4) a global consumer culture with an emphasis on slenderness as a female body ideal. All four of Gordon's factors resonate strongly with Qatar's rapid socio-economic and epidemiological transition. Rates of WBO may therefore be rising concomitantly, with the attendant poor physical, mental, and social health outcomes, including chronic non-communicable diseases such as cardiovascular disease and diabetes. However, no studies have examined any of the concepts related to internalized or external WBO in Qatar. This study aims to examine experiences of external WBO, including teasing, bullying, stigmatization, and discrimination, and the internalized attitudes, values, and beliefs related to body weight in a sample of people in Qatar.

Given the significant body of evidence demonstrating the relationship between population changes in body weight, WBO, and negative health outcomes, it is imperative that research studies begin to explore the full scope of WBO in Qatar and the Arab region. This qualitative study is the first to do so and will provide the foundation for future quantitative studies to examine the extent and impact of these issues in the population more broadly.

## 2. Research design

### 2.1. Research questions

The research questions we explored were 1. What are the internalized WBO related attitudes, values, and beliefs of people in Qatar? and 2. What are the external WBO experiences of people in Qatar?

### 2.2. Methodology

Constructivist epistemology guided this research study, based on the belief that “reality” is socially constructed (49). The consolidated criteria for reporting qualitative research (COREQ) guidelines are used to report the study design and findings (50). This qualitative study used interview methodology (51), which was most appropriate to explore the range of experiences of participants on this issue. Interview methodology provides a deeper and richer understanding of public health issues than purely quantitative methods and is most appropriate where little is already known about the issue, where the issue is sensitive, or where detailed insights are required from individual participants. All these conditions were applicable to exploring WBO in Qatar.

### 2.3. Theoretical framework

The Red Lotus Critical Health Promotion Model (RLCHPM) was used as a theoretical framework for this study (52). The RLCHPM is a modern, holistic, socio-ecological model, that differs from other health promotion models in terms of incorporating a system of values and principles, and applying them in all stages of critical health promotion including community assessment, planning, and implementation, and evaluation (52–54). In the RLCHPM, the pod of the lotus flower represents the holistic health and wellbeing status of people. The stamens of the lotus flower that surround the pod represent the determinants of health and wellbeing related to people's characteristics, including biological, cognitive, affective, and socioeconomic factors and behaviors. The first layer of petals of the lotus flower represents the environmental determinants of health including social, cultural, political, economic, commercial, natural, and built environments. The second layer of lotus flower petals represents the components of the community assessment process. The third, fourth and fifth layers of lotus flower petals represent the components of health promotion program planning, program implementation and program evaluation processes, respectively. The leaves of the lotus plant represent a focus on sustainability. The stem of the plant represents the process of critical reflection. The tuber and roots are the foundation of the plant and represent the values and principles of critical health promotion, including social justice and equity, holistic, salutogenic, and ecosystems conceptions of health and scientific endeavor, allyship and empowerment, beneficence and non-maleficence, and evidence-informed and theory-based practice that are applied across all components of the model. The lotus plant is a dynamic, living organism that exists within a complex ecosystem. All parts of the plant and its environment are interconnected and influence each other (52–54).

This study aimed to investigate WBO as a specific health and wellbeing issue. Understanding the nature and extent of health and wellbeing issues is part of the community assessment phase of the health promotion practice cycle. Using the RLCHPM as the theoretical foundation meant that the study prioritized the participation of people with higher weight (value: priority populations determined by structural inequality), and considered WBO holistically, which meant we were open to the possibility that WBO may have had physical, mental, spiritual, and social health consequences (value: holistic health paradigm), though we did not specifically probe for each aspect. Using the RLCHPM also meant that the study investigated the characteristics of people (stamens) and environments (first petal layer) that contribute to WBO, and how these factors interact and operate at multiple levels from the individual level to the family, community, organization, and society levels (value: systems science).

### 2.4. Research team and reflexivity

The study team (LOH, BAA, BAS) engaged in reflexive practice (55) at weekly meetings, focusing on issues such as adherence to critical health promotion values and principles, as well as quality considerations. This allowed us to examine our own beliefs and assumptions, and think carefully about how these may be influencing our research process, including the risk of prioritizing our own views or opinions. The epistemological position of constructivism meant that the design of the study, data collection and analysis, and interpretation of the findings were all constructed by the researchers and influenced by our personal and professional experiences. LOH is a health promotion academic and practitioner and fat liberation advocate who has been involved in critical fat studies and fat activism in schools, universities, and the community for over 20 years. LOH and BAA have lived experience of external and internalized weight-based oppression, while BAS has witnessed such experiences with family and friends. BAA and BAS were senior undergraduate public health students at the time of the study and became interested in weight-based oppression through interaction and classes with LOH.

### 2.5. Study participants

Participants were recruited from the staff, faculty, and students at Qatar University (QU). Participation was limited to the QU community as this was an exploratory study. The researchers had no significant relationships with most of the participants prior to the study. In the first recruitment phase, participants were known to the researchers as colleagues in other departments or fellow students from other programs. One participant recruited in the first phase was well known to the researchers as a former student. This participant was eager to participate in the study, despite this existing relationship. In the second recruitment phase, the researchers had no relationships with any of the participants prior to the study.

### 2.6. Recruitment and sampling methods

A combination of homogenous and heterogeneous purposive sampling methods were used. All participants shared the common feature of having been exposed to WBO. Following this initial inclusion criterion, heterogeneous sampling was used to maximize variability within the participants. Purposive sampling is one of the most cost-effective and time-effective sampling methods available and is appropriate for studies such as this where the discovery of meaning will benefit from an intuitive approach. In the first phase of recruitment, potential participants with larger bodies were identified through personal knowledge of the researchers. They were informed about the study, and shown pictures illustrating types of WBO including teasing, and discrimination. We asked if they had experienced anything similar, and if so, would they be interested in participating in the study. This method resulted in nine interviews that were conducted in-person. We had not reached data saturation at that point, as new information was emerging with each interview (56), and so we decided to try a new recruitment strategy. Email and QU social media were used to disseminate a poster calling for QU students, staff, and faculty to participate in the study. The poster had the title “Ever been treated badly because of your weight?” and the text read “Teasing, harassment, stigma, and discrimination based on body weight are widespread and cause considerable distress. We are conducting a study exploring how people with a higher body weight are treated by their families, friends, teachers, healthcare providers, the media, and society in general. The study is the first study in the Arab region to explore these issues.” The poster included a cartoon image of a child with a larger body being teased by a group of children. After screening respondents, those that added the most heterogeneity to the sample were contacted and recruited to participate. This recruitment and sampling method resulted in a further 20 completed interviews, at which point data saturation was deemed to have occurred, as no new concepts were appearing in the data (56, 57). Four people initially volunteered to participate in the second recruitment phase but did not respond to requests from the researchers to schedule an interview. The total sample size was therefore 29 participants. This is significantly larger than the range of nine to 17 participants generally required to reach data saturation in studies using qualitative interviews (56), due to the intentional heterogeneity of our sample.

### 2.7. Data collection method

Individual in-depth semi-structured interviews were used to collect the data *via* a set of predetermined but loosely structured questions. Semi-structured interviews enable the comparison of data across participants, but also provide the flexibility to probe or dig deeper on specific issues as appropriate in each individual interview. The first interview was conducted by LOH (MPH, PhD), a female Associate Professor of Public Health with experience in qualitative studies, with BAA and BAS as observers. All subsequent interviews were conducted by BAA and BAS, female senior undergraduate students majoring in public health and trained in research methods and interviewing techniques. The first nine interviews were conducted in person between January and early March 2020. With the participants' permission, interviews were audio-recorded, and field notes were made during and immediately after each interview, including the observations, thoughts, and ideas about the interview. Just after the second recruitment phase was implemented, the COVID-19 pandemic hit Qatar and all on-campus activities were suspended. As such, our interviews moved online and were conducted using WhatsApp, an end-to-end encrypted communication app that is used by almost all residents of Qatar. Depending on the choice of the participant, interviews were conducted digitally *via* WhatsApp video call, audio call, texting or voice notes, or a combination of texting and voice notes, and took place in March and April 2020. All in-person and digital interviews ranged from 30 to 90 min and were conducted in English and/or Arabic according to the participant's preference. Interviews records were transcribed immediately after the completion of the interview and the transcript was provided to the participant to allow for corrections or additions. Transcripts in Arabic were translated into English for data analysis by BAA and BAS, who are native Arabic speakers.

### 2.8. Data collection instrument

The interview protocol (list of questions) was developed in both English and Arabic, and pilot tested with several respondents to establish if the questions were clear and understandable, and to assess if respondents were willing to answer the questions openly and honestly. Changes were made to the interview protocol after pilot testing before use in the study (Supplementary material 1). The translation from English to Arabic was undertaken independently by BAA and BAS and then compared and amended to develop a consensus. Prompts were used by the interviewers to obtain more detailed information.

### 2.9. Data analysis method

The four-step method of analyzing qualitative data was used. This involves preparation of data, data reduction, displaying data, and verifying data (58). Data preparation involved uploading the transcripts to the NVIVO 12 software program (QSR International). Data reduction in NVIVO included line by line coding, looking for similar concepts, grouping concepts into categories, and grouping categories into larger themes. Two researchers (BAA and BAS) independently familiarized themselves with the interview transcripts, recorded initial observations of the data, and identified codes. The analysis used both etic codes, developed based on *a priori* concepts from the interview guide, and emic codes generated from the words of the participants. In the displaying data phase, codes were grouped into categories and categories were grouped into themes. Codes, categories, and themes were discussed by all three researchers (LOH, BAA and BAS) and disagreements were resolved *via* consensus. Data verification was ensured by cross-checking the results with the original transcripts.

### 2.10. Rigor and trustworthiness

To ensure the rigor and trustworthiness of our results, the research design included using a research team and member-checking. In the weekly meetings of the research team held throughout the study, we engaged in researcher reflexivity, with attention to issues related to the quality of data collection, analysis, and interpretation. Working as a research team enabled us to reflect on our preconceived ideas and prevent the imposition of individual ideas or beliefs over those of participants. Member-checking was used to seek the confirmation of study participants regarding their transcripts. Participants were provided with full transcripts from their interview and offered the opportunity to make any amendments they wished, including adding or deleting text. In the results, we used direct quotations from participants to represent their experiences. All strategies added to the trustworthiness and rigor of the research process.

### 2.11. Ethical considerations

This study and its amendments had ethics approval from QU Institutional Review Board (QU-IRB 1070-EA/19). Several ethical issues were carefully considered in this study, including privacy, confidentiality, respect, and non-maleficence. To ensure participants' privacy, in person interviews were conducted in a private venue at Qatar University, and participants were reassured that their personal information would not be made public. Anonymity and confidentiality were ensured by using a pseudonym selected by the participant, and data were secured in a password-protected file on a password-protected computer accessible only by the researchers. Respect was also ensured through informed consent given by the participants prior to conducting interviews, and any ambiguity about the study was explained and clarified. Participants were assured that participation in the study was voluntary and that they had the right to withdraw at any time.

Finally, it is an ethical responsibility for researchers to do no (additional) harm (59). As a research team we carefully considered the issue of non-maleficence, recognizing that participants may have already experienced significant harm because of WBO, and wanting to avoid inadvertently causing any further harm in the recruitment, data collection, data analysis, or reporting processes. Of specific consideration in this study was the possibility for harm resulting from the framing of body weight. Several studies have found that using pathologizing language about body weight is stigmatizing and leads to poor health outcomes (22, 60–63). As such, in this paper, we present these terms in a censored form (ob^*^sity and overw^*^ight) to minimize harm, in accordance with the position taken by researchers, health professionals, and social justice advocates that these terms are slurs (62, 64–66).

We were therefore very deliberate about our decision to not use terms such as “ob^*^se” or “overw^*^ight”. We also chose to not use the term “fat”. Although the word has been reclaimed by fat liberation activists addressing WBO (62), we believe that the term is predominantly regarded as pejorative in the Arab region. As such, in all study materials (including the recruitment email and social media posts, project information sheet, consent form, and interview protocol) and throughout the interviews we adopted the weight-inclusive approach (67) and referred to people as having larger bodies or being at a higher weight (62, 67).

## 3. Results

There were 29 participants from QU faculty, staff, and students (25 females, 4 males), mostly Arabic and born in Qatar, and aged 18–53 years (Table 1). All participants experienced both internalized and external WBO. The study revealed six major themes: characteristics of internalized WBO; nature of external WBO; timing of external WBO; sources of external WBO; extent of external WBO; and the impact of WBO. Each theme included a number of thematic categories. Table 2 summarizes the major themes, categories, and number of participants who experienced each thematic category. Although it was not the aim of the study to quantify the experiences of WBO, the number of participants that spoke about each thematic category is included to provide a sense of the extent of these experiences among the participants.

**Table 1:** Participant characteristics.

**Characteristic**		**Participants**	
		***N*** = **29**	**%**
Gender	Female	25	86
	Male	4	14
Age	Range 18–53 years		
	Mean = 28.3, SD = 10.36		
Student/faculty/staff	Students	20	69
	Faculty	4	14
	Staff	5	17
Duration of stay in Qatar	Born in Qatar	14	48
	20 years or more	3	10
	10– <20 years	4	14
	5– <10 years	1	3
	2– <5 years	4	14
	<2 years	3	10
Marital status	Never married	19	66
	Married	8	28
	Divorced	2	7

**Table 2:** Major themes and thematic categories.

**Major themes**	**Thematic categories**	**Participants**	
		***N*** = **29**	**%**
Internalized WBO	Dissatisfied	21	72
	Sad	21	72
	Embarrassed and ashamed	20	69
	Worthless	10	34
	Frustrated	11	38
	Pathologized labeling	13	45
Nature of external WBO	Bullying and teasing	24	83
	Discrimination	13	45
	Treated badly	7	24
Timing of external WBO	Childhood and teenage years	24	83
	Adulthood	23	79
	After marriage	8	28
Sources of external WBO	Family	27	93
	Friends	15	52
	Media	15	52
	Spouses	8	28
	Healthcare providers	5	17
	Teachers and professors	5	17
	Culture and society	4	14
	People at school	4	14
	People at the gym	3	10
	People on public transport	2	7
	People in public places	2	7
	People in the street	1	3
Extent of external WBO	Normative	22	76
Impact of WBO	Low self-esteem	27	93
	Self-isolation	25	86
	Depression and anxiety	21	72
	Restriction or dieting	19	66
	Bariatric surgery	16	55
	Eating disorders	14	48
	Suicidal ideation or attempt	13	45

In reporting on the themes and thematic categories, representative quotes from the participants are included to illustrate the study's findings. The quotes are provided verbatim, with no amendments to correct for grammar or inclusion of the word “sic” to indicate perceived errors. This is consistent with the recommendation from the Associated Press as described in the Columbia Journalism Review, which states the use of “sic” can be interpreted as “snarky” and giving a sense of “we know better”, at the expense of the quoted source (68). This is particularly important given that English is not the first language of any of the participants in our study. Respecting the words used by participants acknowledges that language is socially constructed, and this approach is therefore consistent with the constructionist epistemology that informs the study. It is also consistent with the health promotion value in the Red Lotus Critical Health Promotion Model of working with people transparently as a culturally and socially sensitive and reflexive ally respectful of all aspects of diversity, as opposed to the selective health promotion practice of working on people as an outside expert without explicit attention to the relevant cultural and social context or all aspects of diversity (52, 54).

### 3.1. Theme 1: Internalized WBO

Research question one for the study was what are the internalized WBO related attitudes, values, and beliefs of people in Qatar? All participants expressed a combination of internalized WBO related attitudes, values, and beliefs including feeling dissatisfied, sad, embarrassed, ashamed, worthless, or frustrated about their larger bodies. Some participants used pathologizing and/or derogatory terms to describe their bodies, indicating internalized WBO related beliefs about the acceptability of using those terms to describe their bodies.

#### 3.1.1. Dissatisfied

Body dissatisfaction was the most common internalized WBO attitude, and the dissatisfaction was long standing. The desire for weight loss and a slimmer body was very strong among participants, leading to a lack of appreciation for their body. Sara 2 expressed the long-term sense of dissatisfaction, saying, “I've just grown up with this idea that I don't look fine and that I weigh too much, and I should have a thinner waist and thinner legs, and all these things mean I don't really appreciate my appearance.” Dani echoed the ongoing sense of dissatisfaction saying, “Every time I look at myself in the mirror, all I see is things I want to change.”

#### 3.1.2. Sad

Participants spoke frequently of how sad they felt about their bodies, and their perceived lack of opportunities as a result of their body size. These opportunities included feeling attractive and feminine, and fitting in with peers. Dana recalled of her adolescent years, “It was horrible, I knew I was overw^*^ight, I knew I looked fat, I couldn't wear stuff that makes me look good, and being a female, I wanted to wear what other girls are wearing.” Participants also spoke about feeling sad but covering it up so that others were not aware of how they were feeling. As Butterfly said, “This doesn't make me feel good, I feel ugly and sad from the inside, but I pretend to be okay.” The tone of participants' voices as they recounted these experiences reflected their words. Participants sounded miserable when they remembered such situations from the past or present and talked about their body in a negative way.

#### 3.1.3. Embarrassed and ashamed

Exposure to external WBO evoked deep feelings of internalized shame and embarrassment for participants. These feelings were still present for many, irrespective of how long ago the WBO occurred. Situations in which shame and embarrassment occurred included being subjected to negative comments by teachers in school, eating meals with family or friends, and eating in public. Aysha said, “I used to feel embarrassed to eat even if I'm hungry, especially in front of people.” Not finding appropriate clothing sizes in stores also led to participants feeling ashamed and embarrassed about themselves, rather than angry at the lack of options available to them. This was compounded by being treated badly by sales assistants, as Meem described, “When it comes to clothes, I feel ashamed and embarrassed when I don't find my size and some of the assistants there laugh at me because of this.”

#### 3.1.4. Worthless

Beyond feeling dissatisfied, sad, embarrassed, and ashamed, many participants expressed feelings of worthlessness. Being around people with smaller bodies increased these feelings due to participants constantly comparing themselves with others, and ascribing judgment about their own relative worth based on their body size. Dani expressed this feeling saying, “I always felt lesser than the people around, less worthy, or less important. I don't think I could ever see myself as a normal, worthy person.”

#### 3.1.5. Frustrated

Many participants expressed feeling frustrated with their bodies and their inability to make themselves smaller or more acceptable. They felt as though they should be able to control their body, lose weight if they tried, but were failing to do so. These feelings of frustration were internalized, and not necessarily shared with others. They also had a significant impact on the wellbeing of participants. Arif explained, “I feel frustrated and angry, but I keep it for myself, and it affects my whole day and my sleep”.

#### 3.1.6. Pathologizing and derogatory self-labeling

About half of the participants used pathologizing terms such as “overw^*^ight” or “ob^*^se” when referring to their own body. In addition, some participants used terms such as “fat” in a negative manner, and other terms that they regarded as derogatory. The use of these labels to describe themselves resulted in more negative feelings and internalized WBO. Meem said, “I look at the mirror and call myself names—fatty, bear, seal—and this makes me feel awful.”

### 3.2. Theme 2: Nature of external WBO

Research question two for the study was what are the external WBO experiences of people in Qatar? External experiences are categorized under the themes of nature, timing, sources, extent, and impact of external WBO. This section addresses the nature of external WBO. All participants were bullied or teased, experienced discrimination, or were treated badly by others because of their larger body size.

#### 3.2.1. Bullying and teasing

Weight-based bullying occurred in different settings and from various people. In addition, bullying and teasing had several forms such as being called names, verbal bullying, and hurtful comments from even the people closest to participants. When recalling such situations, participants were visibly upset or annoyed. Malak vividly recalled one such experience from her time at school, “One time, our teacher brought chocolates to the class, and some girls were taking more than one piece, so I wasn't left with anything. And when the teacher asked why I didn't get chocolate, the girls started saying that ‘she doesn't need chocolate, that is better for her, so she can lose weight' and the other girls started laughing. But I did not do anything and said that I did not take one because I was fasting, although I was not fasting that day.”

#### 3.2.2. Discrimination

Being discriminated against and having limited opportunities when it comes to certain jobs or marriage were very common concerns among participants. Having a larger body was seen as an obstacle between the person and their goals or desires across all aspects of their lives including personal, social, and professional aspects. Sara 2 explained, “In the beginning of my academic life I wanted to get into the psychology major, and I went to speak to the head of department of psychology, so he can tell me if I have a chance. I was speaking to him academically in terms of GPA, courses I've finished, and he said literally “yeah but this is not gonna work, you need to lose weight”. He was like, “we don't have unhealthy people in psychology”. Mustafa described a similar experience of being denied entry to his profession of choice saying, “When it comes to applying for the military medical services, there is a specific weight; if you are higher, they don't accept you.” Discrimination during the school years was equally painful for participants. Malak recalled, “I remember at sports classes when it comes to choosing teams, no one picks me because I am fat and say that I will slow them down.” When sharing these experiences of being discriminated against, participants were very downcast and seemed to feel defeated, especially when talking about losing the opportunity to pursue their dream jobs.

#### 3.2.3. Treated badly

In addition to teasing, bullying, and discrimination, participants were treated badly in other situations because of their larger body size, including at clothing stores, on public transport, at home, and in other public places. Zayed said, “I used to volunteer in animal shelter for dog walks, pet owners were having mean talks with me regarding how will I walk the dog if I cannot walk myself.” Arif felt as though he is treated badly everywhere he goes, explaining, “When I use public transport, and any outside places, everywhere, I feel that I am treated differently compared to others. Like, when I was using the bus, and it was crowded, one man was pushing me and saying that it's okay nothing will happen to me because I have a large body, and that we are not fitting in the bus because of fat people.” Stereotypes about people with larger bodies also resulted in being treated badly. For example, Malak shared an example from her school years, saying “At class when someone smells a bad smell, the girls try to put it on me and give hints that I am the one that smells bad because I am fat and I need to shower a lot, and I don't know where they get this idea from that if you are fat then you smell bad.”

### 3.3. Theme 3: Timing of external WBO

Participants spoke about experiencing WBO at all ages, including in their current lives. However, the experiences that hurt the participants most were those that occurred during childhood and adolescence. Many participants recalled stories from their childhood and school years with great clarity, including the pain felt at the time, and the ongoing shame and embarrassment resulting from the experiences. Participants also shared experiences of WBO in adulthood, and after marriage.

#### 3.3.1. Childhood and teenage years

Most participants were bullied or teased because of their weight as children, and this escalated in teenage years, especially at school. Bullying was experienced from friends, classmates, other students, teachers, and in other settings such as at home, the gym, and other places. Dani vividly recalled her experiences, saying, “I was bullied a lot as a child, especially at school. People I don't know would come up to me and call me names like fatty or bear. They would literally point fingers at me in recess as I walked by them. I remember once being punched by boys because ‘It felt like beating a pillow' they said, and this whole year at middle school they'd call me ‘la vache qui rit' (translation: the laughing cow).” Likewise, Malak described the significant impact of early exposure to WBO, saying, “This affected me since childhood, I always put this idea in my mind that I am fat and this means that I am not like the other girls, and I can't be as beautiful as they are. The effect got even greater when I turned to a teenager. I entered the hospital several times, because of being obsessed with losing weight and looking as good as the other girls without caring about my health or anything else.”

#### 3.3.2. Adulthood

Exposure to external WBO continued into adulthood for most participants. Ongoing exposure was exasperating for some participants, indicating that they may have felt it would decline once they left their childhood years. As Arif said, “This weight bullying started from a young age, and it continued till this time. I am 32 years old, and I am still getting bullied and harassed in different settings? How long will this continue?” Mariam expressed her frustration with ongoing exposure to WBO from her parents, saying, “When we gather as a family to eat, my parents tell me what I am supposed to eat, even though I am a grown a^**^ woman”.

Several female participants spoke about the perception that a larger body is an impediment to getting married. Meem spoke about the dual pressures of men's preferences in a marriage partner, and families' desires to satisfy these, saying “I've also noticed that when it comes to marriage, men usually have a special request that they want to marry a slim and fit woman, with a nice body shape. Family also have a big role... As for my personal experience, my family used to tell me don't get fatter, no one wants to marry a fat woman, and usually hurtful words.”

#### 3.3.3. After marriage

All eight of the married participants experienced significant levels of bullying, teasing, and harassment from their spouses after marriage. This caused significant distress for the participants, which was evident as they described how they are treated differently by their spouse now in relation to their body weight. Maha Mahmoud explained, “I was in that perfect shape in the eyes of society before I get married, after the marriage and you having kids and all of that, my husband started to comment on my body and that I should be losing weight, and that I am not the girl he married with that awful body”.

### 3.4. Theme 4: Sources of external WBO

Participants experienced external WBO from a variety of sources including family, friends, media, spouses, healthcare providers, teachers and professors, culture and society, and people at school, the gym, on public transport, in public places, and in the street.

#### 3.4.1. Family

Most of the oppression based on body weight came from family members, particularly parents. Mothers and fathers put participants under constant pressure to restrict their eating and lose weight. Participants spoke about their parents mocking them because of their high weight or large size and comparing them unfavorably with their smaller sized siblings or others. Parents focused more on the participants' body weight than their achievements. As Dana recalled, “On the day of my master degree graduation, I was the top at my class, honor roll, and when I told my mum ‘Are you proud of me?' she said ‘Yes I am proud, but dear you were the biggest person on stage, you had the highest GPA and the highest weight', and that killed me.” Participants also spoke about being subjected to horrendous shaming experiences by their parents, even as very young children. Dani explained, “I was 7? 8? My biological father would gather my siblings around me as I got up on the scale and would tell them ‘Laugh at your sister' and then would sit me on the dinner table and not let me eat and everyone would call me ‘cow'.”

#### 3.4.2. Friends

Friends were a prevalent source of external WBO, mostly in the form of hurtful comments and making fun of participants about their weight, often pretending they were joking. Participants spoke about the pain and embarrassment that this caused, especially when comments were made in front of others. Malak described one such situation, saying, “When my friends saw my elder sister, they were like ‘She's so much prettier than you are and she looks younger than you. Be more like her and lose weight'.” Malak described the significant hurt that experiences like this caused her. Amal described her perceptions about her relationship with friends, saying, “My friends tend to mock me and tease me because of my weight, and they abandoned me because of my weight, and they were only friends with me because they felt that I'm pathetic.”

#### 3.4.3. Media

More than half of the participants cited the role of the media in perpetuating WBO. This included mass media such as movies and magazines, and social media, particularly Instagram in perpetuating unrealistic ideals about beauty and attractiveness through the use of filters and editing, and through the widespread sharing of before and after weight loss images. Butterfly summed up many participants' beliefs about the presence of models on the platform, saying “Instagram it is full of thin bodies as models, and it is really rare to find a picture of a model with fat body. We have been exposed to role models as thin and slim so it kinda have sticked in our head that the preferred or more beautiful is the thin type of bodies.”

Participants also highlighted the role of media advertising and the selective representation of people with different sized bodies. Gandi explained, “There are advertisements out there that would put someone that's slender in there as if they're running in the forest and it's so beautiful behind them and someone with a large body at home he's sitting, he's depressed he has a bag of chips.” She went on to describe how these images create an association between an image and an emotion, and that such associations may lead some people to believe that this is how they should be. She explained, “I think that actually also affects people watching, so for example, if I would see that person with a larger body which is at home and just sitting and watching TV on their couch and eating chips and they look sad, (I would think) that's probably how I'm supposed to be. And not everyone recognize that's an image, they actually take it in and becomes part of their personality, and that's sad how powerful it (the media) is and how devastating at the same time. It (the media) should be used for good but it's being used for horrible things, really, really, it's so sad.”

Representation of larger bodies in the media as the butt of jokes or as funny characters was also mentioned by participants as a source of negative stereotypes, making them sad and angry. As Sara 2 explained, “It really angers me, because they use their (larger) bodies to make people laugh, instead of using their presence to tell people its normal. We have personalities, rather than seeing us as just a body.”

Sara 2 then described the impact of the combination of sources of WBO on mental health and wellbeing, saying “If it wasn't for the media and pop culture, and how people were raised, and what they see every day of bullying of everything that's different on TV, they wouldn't project it on other people. By constantly joking around not knowing that it can literally put people into depression.” Sara 2 went on to suggest this was a phenomenon confined to the Arabic media, and that “It's really different in American and European media.” Dana also commented on the combined role of the media and the family, saying “Family makes it happen, and then media kind of amplifies it a lot by cartoons and having comics about it, and some drawings in the newspaper of fat people being made fun of. So actually, they create it.”

#### 3.4.4. Spouses

As mentioned in the thematic category about timing, all married participants reported their spouses as a source of external WBO. Participants described how their spouses tease or bully them and monitoring their food intake. As a result, they felt that they are not valued by their spouses and are neglected because of their higher weight. SA explained, “My husband is my number one bully. Can you imagine what it's like to live with a person who teases you and make fun of your body in every situation?” Um Abdulla described a recent situation, saying “Few days ago it was raining, so my husband was praying and saying, ‘Dear god, please let my wife lose weight' as a joke, and I acted like I did not care but it felt bad.” Meem described being called names and having her food intake closely monitored, saying “Even my husband teases me and calls me names such as ‘dubba' (translation: fatty), cow, and seal. And even when I eat, he watches every single bite and tells me ‘You eat a lot'.”

#### 3.4.5. Healthcare providers

Some participants experienced WBO from their healthcare providers. SA described a situation common to several participants, saying, “The first sentence that a doctor would say is your BMI is high, so you need to lose weight. But when you see the other health indicators of me you would see that they are very good and nothing is wrong with me, but when it comes to BMI classification the doctor himself make stereotype for people.” Sara 3 described a similar experience of having every health issue attributed to her weight, saying, “I have regular visits to the hospital because of a certain health issue I suffer from. And each appointment I go to my doctor never forgets to mention how bad it is to be weighing this much, and that maybe all of what I'm in (my health issue) goes back to my weight.” Arif described an experience of having his vital signs assessment conducted by a nurse, saying “When I was around 18 and I got on the scale one time at the hospital, the nurse shockingly told me ‘Omg how could you carry all this weight in one body'.”

Many participants described how being labeled as overw^*^ght or ob^*^se by their healthcare providers made them feel sad or depressed. Meem described the impact of being labeled ob^*^se by her doctor as making her “feel that I'm outcasted or rejected by the society.” She went on to describe her encounters with healthcare providers in relation to pregnancy and giving birth, saying “They always comment on my ob^*^se body. A doctor once told me that because I'm ob^*^se I won't be able to have a baby.” The doctor's bold prediction, rooted in weight bias, was completely inaccurate as Meem went on to have several children.

Health checkups by school healthcare providers were another source of exposure to WBO due to the practice of weighing students and labeling them with the BMI classification. Sara 3 explained her feelings about this, saying “I really don't think these labels are okay because they allow for so much discrimination. Students would wanna compare their results, and for anyone who isn't in the area of ‘ideal weight' or ‘normal weight' it can be such a terrible thing. I don't see the need to do it or put a label on it.”

Not all participants rejected outright the healthcare providers' use of BMI classifications, regarding them as objective and even helpful, whilst paradoxically also acknowledging the negative effect. As Kaltham explained, “If it (the BMI label) is from a nutritionist or a doctor or like trainer then I want to know their opinion… it's a pressure for me to lose weight and exercise and eat healthy food. Okay it would affect me in a negative way but like at the end of the day it would be an incentive way for me to lose weight, do workouts and eat healthy.” Sarah also felt that being labeled with a BMI category would be both helpful and harmful, first saying that BMI categorization is a good thing because it helps people know where they are and what they need to do, and then highlighting the negative impact, saying that if she was labeled as ob^*^se, that “it will affect me, because I'll start to worry about my health.”

#### 3.4.6. Teachers and professors

Teachers and professors were a source of external WBO for some participants. Participants' memories of situations with teachers from school were still intense and vivid, despite many years having elapsed. One of the participants spoke about how, at the age of 45, she could still recall every detail of the weight stigma she experienced from her teacher, how she will never forget how it felt, and the effect it continues to have on her life. Participants associated their experiences of WBO from teachers with loss of productivity, high rates of absence, and low academic performance in school. Butterfly recalled, “I remember once in my school and during the class, I was asking my teacher whether I can turn on the AC (air conditioning) because I was feeling hot, and she replied back to me ‘yeah of course you feel like it's hot because of the fat body' or something like ‘the fat you are carrying'.” The clarity with which Butterfly recalled this exchange with her teacher is indicative of the impact that even brief episodes of exposure to external WBO, particularly for children who are less well equipped to deal with them than adults. Some participants described their negative treatment at the hands of physical education teachers. Dana shared her feelings, saying, “Sports class was one of my least favorite classes. My physical education teacher gave us a test in a certain skill where you jump and flip, and on that day, it was my first menstrual day, so I wasn't able to perform. And when I told my teacher about this, she replied ‘You can just say that you're ob^*^se and ob^*^se people cannot do that'.”

As described above, one participant was prevented from fulfilling her lifelong dream to study psychology due to her professor's belief that people with larger bodies are “unhealthy” and “unhealthy” people cannot be psychologists.

#### 3.4.7. Culture and society

Arabic culture and society were highlighted as significant sources of WBO, with participants noting that this is contrary to the perception outside the Arab world that Arabic culture is more accepting of size diversity. Participants expressed the belief that WBO is regarded as completely acceptable and as a result is widespread in Arabic society. Participants noted that Arabic culture now mirrors western culture, with thin bodies indicating healthiness and large bodies indicating unhealthiness. Having a large body size is widely seen as sign of infertility in the Arab region. Participant also talked about the intergenerational effects, thereby challenging another misconception that WBO is a recent phenomenon in Arabic society. Sara 3 noted, “Our culture and society promotes the idea of big bodies being embarrassing, a problem, and a bad unacceptable thing.” Dana expressed her frustration with the role of culture saying, “What I hate the most about the Arabic culture, is that it always puts the blame on the girl's weight when it comes to everything, whether if she can't get pregnant or not yet married.” Battuta shared her belief that, “A perfect slim body is one of the most cherished ideologies in our Arabic culture, it's like Arab people are programmed to the idea that there is only one perfect body size.”

#### 3.4.8. Others

Sources of WBO were not limited to family members, friends, healthcare providers, or teachers. Participants experienced WBO from other people including people at the gym, school, on public transport, and in streets and other public places. Sara 2 described a situation where strangers felt entitled to dictate her choice of activity, explaining, “Some ladies at the gym were looking at me in a weird way and they later came to tell me that I should do something about myself. I shouldn't be swimming because I shouldn't be wearing a swimming suit. I should go for a walk or something rather than swim, because no one wants to be looking at my body.” Sara 3 described a similar situation in which a stranger felt entitled to make unsolicited comments on her body, saying, “I was once at a conference, and I was wearing a little high waisted pants in which I guess the ‘flaws' of my figure were apparent, and this guy thought it would be okay to poke fun at my figure in the middle of everyone and he made a comment about how I looked ‘a little pregnant'.”

### 3.5. Theme 5: Extent of external WBO

#### 3.5.1. Normative

Although only a small number of participants spoke about the culture as a source of WBO, most participants considered WBO to be an everyday phenomenon in Arab culture. Participants believed that negative attitudes and practices toward people with larger bodies are so extensive and accepted that external WBO is regarded as normative within the Arabic culture. Sara 3 described how extensive this was for her, saying, “I was bullied for being fat in every situation and in every outfit and every celebration, Eid, weddings, etc.” Butterfly commented on the normative and intergenerational nature of external WBO, explaining, “It is has become like a normal thing in the society in our culture, each generation teaches the next one this idea. We grew up knowing that fat bodies are not as acceptable and even that they are shameful, and we can't be in a large size”.

### 3.6. Theme 6: Impact of internalized WBO and exposure to external WBO

Experiencing WBO resulted in negative mental, psychological, emotional, social, and physical consequences such as low self-esteem, self-isolation, depression and anxiety, restrictive eating or dieting, eating disorders, thinking about or proceeding with bariatric surgery, and suicidal ideation or attempt.

#### 3.6.1. Low self-esteem

Almost all participants identified low self-esteem as a consequence of WBO. For participants, low self-esteem encompassed lack of confidence, negative body image, lack of love toward oneself, feeling unworthy and not good enough. For Battuta, “It affected my self-esteem. I started to hate my body and not accepting it. I'm not always at ease when I meet new people and I avoid meeting new people.” Amal explained, “I used to cry, and I hate doing my daily activities. It affected my productivity and sometimes it reached a point where I hurt myself. For example, I used to see myself in the mirror and say ugly! I hurt myself on purpose and I intentionally say that to myself. I really had low self-esteem.”

#### 3.6.2. Self-isolation

As Battuta describe above, low self-esteem resulted in self-isolation. This was common amongst most participants, with exposure to WBO resulting in a range of negative social consequences. Participants spoke about how they actively avoid going out, taking group pictures, being around people or being socially engaged, especially on special occasions or at gatherings of family and friends. Aysha explained, “I became a person who don't want to be engaged in the community. I used to feel really shy and embarrassed, so I isolated myself from people.” Hala described a similar strategy, saying, “I isolated myself. I used to not want to be friends with anyone. I preferred to be alone to avoid people's comment. I used to hate going to occasions, and if I ever go, I used to stay in abaya because I hated how clothes look on me.” SA explained the impact on her, saying, “I hated going out to see people, I hated gathering with people. I don't approach people and talk to them because of my weight. So, it affected me socially a lot.”

#### 3.6.3. Depression and anxiety

Participants strongly believed that WBO resulted in mental health conditions, including depression and anxiety requiring professional treatment. Dani described the impact for her, saying, “I suffered from depression for years and I used to be on antidepressants, till now I have depressive episodes from time to time. You hate your body, you hate yourself, you hate them as well. The psychological effect that this led to is much more serious than the weight itself.” Sara 2 also highlighted that these effects were because of exposure to WBO, explaining, “I would just say that I do not suffer from any health issues because of my weight. The only issues I have are on my mental health, anxiety and depression and these are not because of my weight they are because of how people view my weight and body”.

#### 3.6.4. Restriction and dieting

Two-thirds of the participants spoke about their repeated attempts to change their body weight and thereby escape WBO through restricting their eating or embarking on diets. Many of these diets were deficient in nutrients, and unsustainable over a sustained period. Battuta recalled one such diet, saying, “I remember one time, I tried the ‘watermelon diet', so it basically tells you to eat nothing but a watermelon for a certain time, and I experienced horrible weight and hair loss at that time.” Zayed discussed the range of diets he had attempted, and the damaging consequences for his relationship with food, saying “I tried every weight loss method that can come across your mind. I reached a point where I fear food and count calories for every single food item I consume.”

#### 3.6.5. Eating disorders

Almost half of the participants described how exposure to WBO led to the development of disordered eating behaviors and eating disorders, including self-induced vomiting, binge eating, emotional eating, and bulimia. Malak described her situation, saying, “I felt like I craved food more and more till it turned to binge eating. I started to eat without stopping, till I slowly reached my previous weight, and that made me commit a very awful thing, which was eating and then putting my finger in my mouth to induce vomiting, and this habit stayed with me for almost a week and then my body could not handle it anymore, so I went to the hospital.” Dani described how she developed an eating disorder as a child, saying, “I felt like this is always something that I had to focus on. I can't remember a single time period in my life that my weight obsession was out of the picture. It led to me forming bulimia at the age of 12.”

#### 3.6.6. Bariatric surgery

Over half of the participants considered or had undergone bariatric surgery because of exposure to WBO, including sleeve gastrectomy, adjustable gastric band, and gastric balloon. Although this surgery has a high level of risk, it was seen as an acceptable and almost routine procedure to reduce exposure to WBO. As K stated, “Qatari society has one preference to the point that anyone with extra kilos will be told ‘there are surgeries to cut some weight, go have one'.” Dana talked about the process that led up to her decision, explaining, “I said ‘Well I'm sick and suffering, people still see that I'm fat, they don't see my achievement, so you know what, let me do this as a last resort. I'll do the gastric sleeve operation and see what happens'.”

#### 3.6.7. Suicidal ideation or attempt

One of the most serious impacts of experiencing WBO was thinking about or attempting suicide. Almost half of the participants expressed wanting to end their lives at some point to relieve their suffering from exposure to WBO. Whilst recalling these feelings, participants demonstrated deep sorrow, and some were crying while sharing these experiences. Hala shared her experience, saying, “I had depression from time to time and I was always thinking of a way of dying, and I knew that if I confessed this to my family, they will not take it seriously. So I was lonely, and I attempted suicide using pills and went to hospital. At that time, I had my son who was 8 years old.” Butterfly shared her story, saying, “I couldn't take it any further, I even thought of ending my life instead of living this every single day.”

## 4. Discussion

This study explored experiences of weight-based oppression experiences among 29 students, staff, and faculty at Qatar University. WBO was perceived to be so common that it was regarded as normative and intergenerational. Experiences of WBO included teasing, harassment, stigmatization, and discrimination based on body weight from family, friends, spouses, healthcare providers, teachers, and other people. The media and Arabic culture were also regarded as important sources of WBO. Experiences of WBO occurred throughout life, but those that occurred in childhood and adolescence were particularly painful. These experiences had significant and lasting negative psychological, emotional, social, and physical consequences for participants' health and wellbeing. Participants experienced negative internalized feelings, beliefs, and attitudes about their own body weight such as low self-esteem, embarrassment, shame, body dissatisfaction, sadness, and worthlessness. Exposure to WBO resulted in social isolation, depression and anxiety, food restriction, dieting, disordered eating, and eating disorders. Some participants had thought about or tried bariatric surgery or suicide to escape WBO.

This is the first study to qualitatively explore experiences of weight-based oppression in Qatar and the Arab region, and in many respects, the study findings are comparable to those from studies in other regions of the world. In this study, participants perceived WBO to be highly prevalent in Qatar and the Arab region. In fact, WBO is perceived as being so common that it is regarded as normative and completely acceptable. This perception is consistent with studies elsewhere that have demonstrated the high prevalence of various forms of WBO such as teasing, bullying, and discrimination in countries in the Global North (69). Further population-based studies are needed to determine if the actual prevalence of WBO is as high in the Arab region as perceived.

Participants experienced WBO at all ages, but the impact of exposure during childhood and adolescence was particularly significant. Sources of WBO at a young age included parents, friends, and teachers. Young people with higher body weight are vulnerable to weight-based bullying, harassment, stigmatization and teasing in school settings (3, 70). Weight-based bullying is one of the most common types of bullying that children and youth face (71–73). Children and adolescents are mostly frequently exposed to WBO from family, friends, peers, and teachers. The memories of these exposures to WBO for participants in our study were very strong, and many years later evoked significant sadness. Although participants recalled many experiences WBO at other times in their lives, it was these childhood exposures that they seemed to be particularly damaging. Children are less equipped to deal with exposure to hurtful comments or behaviors, and if the perpetrators of those behaviors are people in positions of trust and authority, then the capacity of young people to reject these behaviors and resist the internalization of the messages is limited. Particular attention should therefore be paid to eliminating WBO in the structures and systems that most impact on young people, such as the family and school environments.

In adulthood, sources of WBO for our participants included family, friends, professors, healthcare providers, and for married participants, spouses. This is consistent with findings from other studies demonstrating the extent of this issue. WBO from family was the most commonly reported exposure, and participants relayed painful examples of their treatment at the hands of their parents and siblings. All married participants shared devastating experiences of WBO from their spouses. WBO from family can be the most painful experience as it comes from people who supposedly love and protect you (74). With respect to healthcare providers, our participants spoke about the healthcare inequalities they have experienced from doctors and nurses, which reflects a significant body of literature demonstrating high levels of WBO from these professions. A recent systematic review confirmed widespread weight bias in a range of healthcare providers (75).

Interestingly, only four of our participants identified Arabic culture and society as a source of WBO, however almost all described WBO as being universal within Arabic culture. This differential may reflect the widespread belief that WBO is totally “normal”, and that is it not specific to Arabic culture. Various studies have investigated aspects of WBO such as weight bias internalization, exposure to weight stigmatizing experiences, and weight discrimination in the Global North, with most of these studies conducted in the USA (76). Investigations in the Global South and in the Arab region in particular, are limited. More broadly, the cultural imperialism of the Global North has seen the adoption of “western” appearance ideals, particularly the thin/non-fat ideals for all genders. Rates of eating disorders have rapidly increased in the Global South (77) and identification with western culture is associated higher levels of eating disorder symptomatology for Arabic women (78). The role of mass media and social media in this messaging was well recognized by our participants, consistent with literature demonstrating strong links between exposure to media and WBO in western cultural contexts (79).

Turning to the consequences of WBO, our study found that exposure to WBO results in significant psychological distress, including shame, embarrassment, feelings of worthlessness, depression, and anxiety. Many participants demonstrated significant levels of internalized WBO, indicating their belief that there is something inherently wrong with their larger bodies. Some participants also identified that their psychological distress was because of their unfair treatment based on their body weight. The strong relationship between psychological distress and WBO is consistent with many other studies. A recent systematic review (80) and meta-analysis (81) established that exposure to WBO is consistently associated with depression.

One of the most common behavioral consequences of exposure to WBO is changes to eating patterns. In our study we found that participants reported engaging in food restriction and dieting in response to internalized and external WBO. For many, the adoption of food restriction or dieting was strongly encouraged or even demanded by parents or family members. However, there is now a significant body of research that demonstrates the failure of dieting to sustainably reduce body size (82, 83), and subsequent weight regain is often attributed to the failure of the dieter, rather than a natural physiological response to dieting, furthering the shame that people feel about their bodies. For some participants in our study, restriction and dieting escalated into eating disorders, and others discussed developing eating disorders as a direct result of exposure to WBO. A recent scoping review revealed that rates of eating disorders in the Arab region averaged 31% with some studies detecting rates up to 75% in specific samples (84). The relationship between internalized WBO, exposure to WBO, disordered eating, and eating disorders is well documented (85, 86). Within the Arab region, studies on males in Kuwait (87) and females in the United Arab Emirates (14) have found associations between internalized or external WBO and eating disorder symptomatology. This is consistent with other studies that have demonstrated that weight-based stigmatization is associated with a range of behavioral consequences, including binge eating disorder (88). Weight-based teasing in adolescence prospectively predicts binge eating up to 5 years later (89, 90).

Our finding that exposure to WBO resulted in suicidal ideation and suicide attempts adds to the existing literature about the severe consequences of WBO. WBO is associated with higher levels of suicidal ideation in adults, with the effect mediated by depression (91). Likewise for young people (92) and children (93), the most serious emotional consequence of WBO is the increased risk of thinking about and attempting suicide. Adolescents who are teased about their body weight are two to three times more likely to have suicidal thoughts than those not subjected to such teasing (94). In this study, around half of the participants had suicidal ideation or suicide attempts. This is consistent with the findings of a study that found over 50% of females and 13% of males who were exposed to WBO from their family and friends considered attempting suicide (9). This is hugely concerning, and further research is urgently required to establish the prevalence of such consequences in Qatar and the Arab region.

The decision to undertake bariatric surgery such as sleeve gastrectomy, adjustable gastric band, and gastric balloon was another consequence of being exposed to WBO, and more than half of the participants in this study were considering such surgery or had already had it. This was particularly the case for participants who expressed feelings of sadness and depression, or that had disordered eating or eating disorders. This finding was expected as eating disorders, anxiety, and depression are prevalent in candidates for bariatric surgery (95), with depression and eating disorders more prevalent in bariatric surgery candidates than in the general population (96). Undergoing bariatric surgery also increases the subsequent risk of self-harm, suicidal ideation, suicide attempts, and suicide (97, 98). Making the choice to undergo bariatric surgery to escape WBO and its psychological sequalae is understandable, given the extremely low efficacy of other weight loss strategies, the internalization of negative beliefs and attitudes about higher weight, and ongoing exposure to the stress of WBO. Bariatric surgery is widely practiced in Qatar, and although no official government statistics are available, news reports stated that in 2014, around 2,000 surgeries were performed in a population at the time of 2.2 million people (99), with 70% of recipients being women. This was compared to the rate in Japan where 200 bariatric surgeries were performed in a population of 127 million. In 2022, the government health service reported that it performs 800–1,000 surgeries per year (100). The normative nature of WBO combined with the normalization of bariatric surgery creates the perfect storm to drive up rates of bariatric surgery.

This study explored WBO using the RLCHPM as a theoretical foundation. Exploring and understanding a health issue is part of the community assessment phase, which is the first phase in the health promotion process. By conducting regular critical reflection (represented by the stems of the plant in the RLCHPM) on the values and principles in the RLCHPM (represented by the tuber and roots), we ensured that the study explored WBO holistically and revealed a range of physical, mental, and social consequences of WBO (represented by the lotus flower pod). In addition, the RLCHPM guided us to examine the characteristics of people (represented by the stamens of the lotus flower) and different types of environments (represented by the first petal layer of the flower) that lead to WBO, and how these factors connect and operate at multiple levels from the individual level to the family, community, organization, and society levels. Using the RLCHPM therefore ensured that we took a socio-ecological or systems approach to the exploration of WBO (a value and principle represented by the tuber and roots). Finally, critically reflecting on the potential for harm throughout the research process helped us to minimize potential harms (a value and principle represented by the tuber and roots).

The findings from this study indicate that WBO operates at the intrapersonal, interpersonal, family, community, society, and population levels, and has significant negative psychological, emotional, behavioral, social, and physical consequences resulting in poor health outcomes. None of the participants indicated that WBO resulted in improvements in their health and wellbeing. This is contrary to the commonly held belief, also expressed by some public health writers, that greater exposure to WBO might give people with higher body weight the “motivation” to improve their (assumed) poor health and change their (assumed) poor behaviors (101). This study provides evidence that such an approach would not only be unsuccessful at such “motivation”, but would perpetuate and extend the harm caused by WBO.

### 4.1. Strengths and limitations

There are several strengths of this qualitative exploratory study. It provides the first insight into the lived experiences of WBO of people in the Arab region. The study provides a holistic view of the phenomenon with exploration of participants' experiences of WBO from many different perspectives and at multiple inter-related levels. As a method for exploring a sensitive topic, using semi-structured interviews allowed participants to express themselves in their own words, and share their experiences in as much detail as they wished. In-person and WhatsApp call interviews also allowed for the observation of the body language and speaking tone of participants, and to connect their emotions to their words. Although the requirement to switch to online interview administration was initially regarded as a limitation potentially impacting on the quality and quantity of data, it was apparent that this may have inadvertently had a positive impact. We noticed that participants who used text or voice notes to complete their interviews provided significantly longer and more in-depth responses to the interview questions than many of those interviewed in person or *via* a WhatsApp video or audio call. This observation warrants further research to validate if using text and/or voice note exchanges provide the same rigor and quality as other more established interview administration methods.

Despite the important contributions of this study's findings, limitations must be considered. A limitation of the single face-to-face interview is that there was little time to build trust and rapport with participants. Because the interview addressed a sensitive issue and one that involves significant pain for many participants, the limited time may have inhibited their responses. A second limitation was the discrepancy between the interviewers and participants' body sizes. Neither of the interviewers is fat, and this may have affected the responses provided by participants in the in-person interviews. This may not have impacted on the interviews conducted *via* text or voice notes. A third limitation of the study is that the findings were generated from a relatively small group of people within the Qatar University community. Further studies are required to determine if these experiences are similar or different to those of people in the broader community in Qatar and the Middle East, and the extent of this issue in the Arab region. Finally, the findings from this study are particular to the participants and the interpretation of the researchers.

## 5. Conclusion

WBO in the Arab region is an important and unrecognized public health issue. This study, the first of its kind in the Arab region, demonstrated that WBO is so common that it is regarded as normative. For participants in our study, WBO had significant negative implications for their physical, mental, and social health and wellbeing. Further research is required to determine the nature and extent of WBO within the broader community and other countries in the Arab region. In addition, research must be conducted to develop and test the effectiveness of critical health promotion strategies to reduce internalized and external WBO in all sectors. Critical health promotion involves addressing systemic and structural sources of oppression (52) using a portfolio of strategies encompassing building healthy public policy, creating supporting environments, and strengthening community action as priority strategies. Of particular urgency is the need to develop critical health promotion programs addressing social and cultural systems and structures to reduce teasing, bullying, and negative experiences related to body weight in childhood. This will require working with governments, social media, corporations, parents, teachers, healthcare professionals, young people, and the community to develop critical health promotion programs that reduce children's exposure to toxic messages about their bodies and weight-related practices that are harmful to them.

## Data availability statement

The raw data supporting the conclusions of this article will be made available by the authors, without undue reservation.

## Ethics statement

This study involving human participants was reviewed and approved by Qatar University Institutional Review Board. The participants provided their written informed consent to participate in this study.

## Author contributions

LO'H conceived and designed the study. BAA and BAS collected the data. All authors analyzed the data, wrote the manuscript, contributed to manuscript revision, read, and approved the submitted version.
