# Supplementary material for: “I was bullied for being fat in every situation, in every outfit, at every celebration”: A qualitative exploratory study on experiences of weight-based oppression in Qatar
Source: Front Public Health. 2023 Feb 27;11:1015181. doi: 10.3389/fpubh.2023.1015181 (PMC10008867; doi:10.3389/fpubh.2023.1015181)
Supplement: Supplementary file 1 [file Table_1.DOCX]

**Experiences of weight-based oppression in Qatar residents**

**Interview Protocol**

Researcher: Thank you for agreeing to participate in this study. It is important for me to be able to concentrate on what you are saying, and so I would like to request your permission to record our interview so that I can be sure not to miss anything you’re saying, and to capture your experiences in own words. As explained in the consent form, the digital recording will be transcribed into a Word document and will then be deleted from all devices. The transcripts will be kept in password protected files on password protected computers and your identity will never be disclosed. Do I have your permission to record the interview? *(Ensure participant gives permission verbally not just by head gesture.)*

1. Could we begin by asking you to name a pseudonym that you would like to be known as in the results. *(Record pseudonym on the top of the demographic questionnaire. Ensure no pseudonym is used by two different people.)*
2. I’d like to ask you to talk about how you feel different body shapes and sizes are regarded here in Qatar. Are some shapes and sizes valued more than others?
3. What do you think are the reasons for this? Probes: role of family, friends, culture, media
4. How have these factors or beliefs affected you personally? Probes: self-esteem, self-belief, restrictive or disordered behaviours, limited opportunities
5. How do you feel about your own body weight? Why do you think you feel this way?
6. *If the participant uses a medicalized category for their body weight (eg. underweight, healthy weight, overweight etc.):* What do you think that label or category means? How does using that label make you feel? Do you feel as though it is helpful to you? Do you think it is helpful to people generally to be labelled like that?
7. Do you ever talk about your body in a negative way? Tell me about this. How does it make you feel?
8. Have you ever felt sad, frustrated or angry when someone talks about body weight in a negative way? Can you tell me more about this?
9. Do you think you have ever been treated badly because of your weight? Can you tell me more about this? In what setting has this happened? How did this make you feel? How often has this happened to you?
10. Have you ever been in a situation in which you have been teased or bullied because of your weight? Can you tell me some more about that? How did you react to that? How often has this happened to you?
11. Can you recall a time you were discriminated against because of your weight? Can you tell me some more about that? How often has this happened to you?
12. In which of your social circles do you usually experience any of these negative treatments based on your body weight?
13. Do you see people with your body type in the media? How does this make you feel?
14. Have you ever heard the term “body positive”? What does it mean to you?
15. Body positivity means valuing your own and other people’s unique identities and working towards liberation from self-hatred and weight-based oppression for people of all shapes and sizes. Do you ever talk about your body in a positive way? Tell me about this. How does it make you feel?
16. Do you have any other comments you’d like to make about how you feel about your own body, or the way people are treated based on their body weight?
17. To help us identify patterns or similarities in the stories people tell us in this study, we would also like to gather some demographic information from you. Can you please complete these demographic questions. *(Provide the demographic questionnaire and ensure that the participant’s pseudonym has been included.)*

*(After the questionnaire has been returned to the researcher)*

Researcher: Thank you so much for your time and for sharing your story with us. We will provide the transcript of the interview to you once it is completed. You will have the opportunity to make any corrections or amendments you’d like to make before sending it back to us. At the end of the study, we will provide you with a short summary of the overall findings.
